# Supplementary material for: Porcine Reproductive and Respiratory Syndrome Virus Interferes with Swine Influenza A Virus Infection of Epithelial Cells
Source: Vaccines (Basel). 2020 Sep 5;8(3):508. doi: 10.3390/vaccines8030508 (PMC7565700; doi:10.3390/vaccines8030508)
Supplement: Supplementary file 1 [file vaccines-08-00508-s001.pdf]

Table 1. Statistical comparison between mRNA relative expressions in swIAV H1N2 (I) single infections and co-inoculations of NPTr cells with PRRSV-1 (P). The mean values and the standard deviations of the relative expression are shown for every condition. *P* values are presented in the last column. Comparisons were made using one-way ANOVA test and Tukey's post-test.

	Messenger RNA	mRNA relative expression ± SD				I24 vs. I24_P24
		Control	P24	I24	I24_P24	
Pattern recognition receptors	**RIG-I**	1.94 ± 0.1	2.04 ± 0.3	14.83 ± 2.5	2.48 ± 0.9	<0.0001**
	**MDA5**	2.09 ± 0.3	1.71 ± 0.2	4.50 ± 0.7	1.97 ± 0.7	0.0009**
	TLR2	3.72 ± 1.6	4.49 ± 2.4	6.99 ± 5.8	3.15 ± 3.1	ns
	TLR3	2.78 ± 0.4	2.23 ± 0.3	1.92 ± 0.3	1.62 ± 0.4	ns
	TLR4	2.45 ± 2.0	4.26 ± 4.9	3.15 ± 4.8	3.51 ± 3.8	ns
	TLR6	6.80 ± 2.0	6.02 ± 1.4	3.42 ± 0.6	3.22 ± 1.4	ns
	TLR7	2.65 ± 0.9	2.01 ± 0.6	2.01 ± 0.6	1.92 ± 0.8	ns
	TLR8	4.90 ± 5.2	9.41 ± 12.6	15.46 ± 22.1	27.56 ± 40.2	ns
	TLR9	9.83 ± 15.9	23.21 ± 16.8	19.31 ± 9.5	20.49 ± 20.7	ns
	TLR10	4.20 ± 1.2	2.68 ± 1.5	1.50 ± 0.3	3.63 ± 2.9	ns
Interferons	IFNα	3.56 ± 1.1	4.25 ± 1.8	3.88 ± 0.8	5.45 ± 4.5	ns
	**IFNβ**	21.88 ± 17.4	42.25 ± 19.7	3911.05 ± 726.6	673.17 ± 195.4	0.0011**
	IFNλ1	2.27 ± 0.9	2.75 ± 1.1	5.31 ± 1.0	3.53 ± 1.5	ns
Interferon stimulated genes	**PKR**	1.87 ± 0.2	2.23 ± 2.0	4.74 ± 2.1	1.96 ± 0.9	0.0392*
	**OAS**	59.25 ± 6.9	74.35 ± 21.3	1677.44 ± 373.0	100.67 ± 69.9	0.0015**
	**MX1**	1.57 ± 0.2	1.49 ± 0.2	22.04 ± 4.6	2.75 ± 0.8	0.0031**
	**MX2**	6.46 ± 1.4	6.96 ± 1.4	61.01 ± 11.0	6.44 ± 3.4	0.0001**
	ISG12	47.60 ± 15.2	66.88 ± 18.3	111.00 ± 60.1	48.99 ± 43.9	ns
	**ISG15**	2648.03 ± 1365.5	5241.87± 1400.4	209624.87 ± 54923.2	22576.62 ± 1243.8	0.0003**

Ns: not significant, SD: Standard deviation, ***P*<0.01 and **P*<0.05, Genes showing significant differences are represented in bold.

Table 2. Statistical comparisons between mRNA relative expressions in swIAV H1N2 (I) single infections and co-inoculations of NPTr cells with PRRSV-1 (P) with 1 and 6 hours' delay. The mean values and the standard deviations of the relative expression are shown for every condition. *P* values are presented in the last four columns. Comparisons were made using one-way ANOVA test and Tukey's post-test.

	Messenger RNA	mRNA relative expression ± SD									I23 vs. P24_I23	I18 vs. P24_I18	I24 vs. I24_P23	I24 vs. I24_P18
		Control	P24	I18	I23	I24	P24_I23	P24_I18	I24_P23	I24_P18				
Pattern recognition receptors	**RIG-I**	1.94 ± 0.1	2.04 ± 0.3	16.79 ± 2.87	19.89 ±5.82	14.83 ± 2.5	6.13 ± 3.62	9.18 ± 2.96	21.36 ± 8.4	28.02 ± 5.4	0.0079**	ns	ns	0.0486*
	**MDA5**	2.09 ± 0.3	1.71 ± 0.2	5.28 ± 0.23	5.75 ± 1.42	4.50 ± 0.7	3.31 ± 0.88	3.78 ± 1.13	5.75 ± 1.9	8.44 ± 1.3	ns	ns	ns	ns
	TLR2	3.72 ± 1.6	4.49 ± 2.4	8.72 ± 3.3	6.51 ± 3.82	6.99 ± 5.8	7.77 ± 3.95	7.45 ± 5.65	6.76 ± 2.5	8.08 ± 5.7	ns	ns	ns	ns
	TLR3	2.78 ± 0.4	2.23 ± 0.3	2.63 ± 0.24	2.13 ± 0.54	1.92 ± 0.3	2.62 ± 0.57	2.94 ± 0.6	2.31 ± 0.2	2.31 ± 0.4	ns	ns	ns	ns
	TLR4	2.45 ± 2.0	4.26 ± 4.9	1.57 ± 2.44	2.08 ± 1.41	3.15 ± 4.8	4.26 ± 6.36	4.79 ± 3.44	6.43 ± 4.5	1.53 ± 1.6	ns	ns	ns	ns
	**TLR6**	6.80 ± 2.0	6.02 ± 1.4	3.03 ± 1.17	3.52 ± 0.29	3.42 ± 0.6	7.6 ± 1.89	7.05 ± 1.8	4.27 ± 0.6	4.47 ± 1.5	0.0013**	0.0004**	ns	ns
	TLR7	2.65 ± 0.9	2.01 ± 0.6	2.54 ± 0.46	2.01 ± 0.6	2.01 ± 0.6	2.01 ± 0.6	2.01 ± 0.6	2.80 ± 0.3	2.76 ± 0.4	ns	ns	ns	ns
	TLR8	4.90 ± 5.2	9.41 ± 12.6	9.35 ± 6.56	10.96 ± 9.64	15.46 ± 22.1	86.97 ± 62.99	20.39 ± 21.46	14.43 ± 20.1	4.43 ± 8.8	ns	ns	ns	ns
	TLR9	9.83 ± 15.9	23.21 ± 16.8	36.25 ± 13.4	28.91 ± 13.8	19.31 ± 9.5	21.34 ± 8.78	19.87 ± 10.09	9.12 ± 5.9	18.07 ± 12.0	ns	ns	ns	ns
	**TLR10**	4.20 ± 1.2	2.68 ± 1.5	2.03 ± 1.01	2.28 ± 0.65	1.50 ± 0.3	4.77 ± 1.81	5.15 ± 2.01	2.73 ± 0.6	2.20 ± 1.6	0.0448*	0.0062**	ns	ns
Interferons	**IFNα**	3.56 ± 1.1	4.25 ± 1.8	3.62 ± 0.89	2.97 ± 0.41	3.88 ± 0.8	5.82 ± 1.69	4.47 ± 2.05	2.77 ± 0.5	2.62 ± 0.7	0.0317*	ns	ns	ns
	**IFNβ**	21.88 ± 17.4	42.25 ± 19.7	4974.1 ± 858.5	7334.6 ± 2013.14	3911.05 ± 726.6	1897.06 ± 1072.05	3384.59 ± 216.18	5237.35 ± 1460.9	8161.37 ± 1662.9	0.006**	ns	ns	0.0097**
	IFNλ1	2.27 ± 0.9	2.75 ± 1.1	5.97 ± 1.92	6.12 ± 1.94	5.31 ± 1.0	4.33 ± 1.10	5.78 ± 1.71	5.68 ± 1.5	8.71 ± 2.2	ns	ns	ns	ns
Interferon stimulated genes	**PKR**	1.87 ± 0.2	2.23 ± 2.0	6.52 ± 0.69	7.72 ± 0.68	4.74 ± 2.1	3.10 ± 1.45	4.84 ± 0.98	5.91 ± 2.2	6.24 ± 3.6	0.0026*	ns	ns	ns
	**OAS**	59.25 ± 6.9	74.35 ± 21.3	2302.61 ± 442.6	2409.17 ± 625.62	1677.44 ± 373.0	293.18 ± 169.09	640.35 ± 132.64	2034.66 ± 508.0	4931.54 ± 1228.9	0.0004**	0.0046**	ns	0.0067**
	**MX1**	1.57 ± 0.2	1.49 ± 0.2	27 ± 2.89	32.27 ± 7.22	22.04 ± 4.6	7.28 ± 3.57	12.12 ± 1.95	32.67 ± 7.8	53.08 ± 11.8	0.0054**	ns	ns	ns
	**MX2**	6.46 ± 1.4	6.96 ± 1.4	76.60 ± 9.57	104.36 ± 24.31	61.01 ± 11.0	17.34 ± 8.96	34.70 ± 6.05	92.21 ± 28.5	159.12 ± 37.5	0.0002**	0.0436*	ns	0.0229*
	**ISG12**	47.6 ± 15.2	66.88 ± 18.3	138.32 ± 29.34	141.56 ± 26.67	111.00 ± 60.1	74.99 ± 49.32	78.26 ± 38.43	117.07 ± 26.1	147.15 ± 35.6	0.0292*	ns	ns	ns
	ISG15	2648.03 ± 1365.5	5241.87± 1400.4	244759.59 ± 144055.18	272094.50 ± 65321.64	209624.87 ± 54923.2	80336.62 ± 83086.94	98097.28 ± 20568.71	226895.23 ± 163975.2	682603.42 ± 230651.7	ns	ns	ns	ns

Ns: not significant, SD: Standard deviation, ***P*<0.01 and **P*<0.05, Genes showing significant differences in one of the conditions at least are represented in bold.

Table 3. Statistical comparisons between mRNA relative expressions in swIAV H1N2 (I) single infections and co-inoculations of NPTr cells with active (P) and inactivated PRRSV-1 (Pinac). The mean values and the standard deviations of the relative expression are shown for every condition. *P* values are presented in the last three columns. Comparisons were made using one-way ANOVA test and Tukey's post-test.

	Messenger RNA	mRNA relative expression ± SD					I24 vs I24_P24	I24 vs I24_Pinac	I24_P24 vs I24_Pinac
		Control	P24	I24	I24_P24	I24_Pinac			
PRRs	**RIG-I**	2.79 ± 0.9	3.91 ± 0.5	105.93 ± 26.1	1.38 ± 0.2	4.70 ± 1.2	<0.0001 **	0.0004**	<0.0001**
	**MDA5**	2.32 ± 0.2	2.14 ± 0.2	13.87 ± 0.8	1.51 ± 0.3	2.69 ± 0.5	<0.0001 **	ns	<0.0001**
Interferons	**IFNβ**	9.39 ± 3.2	8.46 ± 3.9	665.89 ± 116.5	9.29 ± 9.3	26.78 ± 6.5	<0.0001 **	ns	0.0006 **
	**IFN λ1**	225.87 ± 49.1	29.85 ± 21.4	780.17 ± 228.9	27.64 ± 3.9	75.39 ± 62.8	<0.0001**	<0.0001**	ns
	**IFNλ3**	32.55 ± 37.9	166.08 ± 78.6	30523.76 ± 9265.3	277.79 ± 64.6	938.36 ± 372.4	<0.0001**	0.0074**	<0.0001**
Interferon stimulated genes	**PKR**	5.30 ± 0.8	3.92 ± 0.6	30.70 ± 3.5	1.15 ± 0.1	5.36 ± 0.8	<0.0001**	<0.0001**	<0.0001**
	**OAS**	1.57 ± 0.5	18.01 ± 3.2	434.40 ± 109.1	4.43 ± 0.9	23.18 ± 8.2	<0.0001**	<0.0001**	<0.0001**
	**MX1**	2.54 ± 0.6	4.42 ± 0.8	164.11 ± 28.5	1.45 ± 0.3	8.74 ± 2.3	<0.0001**	0.0070**	<0.0001**
	**MX2**	6.62 ± 1.2	10.64 ± 2.9	527.58 ± 87.1	1.67 ± 0.4	15.54 ± 3.8	<0.0001**	0.0001 **	<0.0001**
	**ISG12**	2.78 ± 0.3	1.79 ± 0.2	6.32 ± 0.7	1.50 ± 0.2	2.90 ± 0.4	<0.0001**	0.0180*	<0.0001**
	**ISG15**	1.64 ± 0.3	7.35 ± 1.5	250.15 ± 118.5	8.57 ± 1.6	17.95 ± 5.8	<0.0001**	ns	0.001**

Ns: not significant, SD: Standard deviation, ***P*<0.01 **P*<0.05, Genes showing significant differences in one of the conditions at least are represented in bold.

N s : n o t s i g n i f i c a n

Table 4. Statistical comparisons between mRNA relative expressions in swIAV H1N2 (I) single infections and multiple infections of Precision-Cut Lung Slices (PCLS) with active (P) and inactivated PRRSV-1 (Pinac). The mean values and the standard deviations of the relative expression are shown for every condition. *P* values are presented in the last three columns. Comparisons were made using one-way ANOVA test and Tukey's post-test.

	Messenger RNA	mRNA relative expression ± SD					I24 vs I24_P24	I24 vs I24_Pinac	I24_P24 vs I24_Pinac
		Control	P24	I24	I24_P24	I24_Pinac			
PRRs	**RIGI**	20.23± 29.5	24.55 ± 12.5	21647.0 ± 5685.1	31.14 ± 22.7	49.87 ± 22.4	<0.0001 **	0.0057**	ns
	**MDA5**	69.12 ± 97.6	39.05 ± 30.4	2634.07 ± 7029.9	21.61 ± 12.1	75.96 ± 54.8	<0.0001 **	ns	ns
Interferons	**IFNβ**	1.37 ± 1.5	1485.37 ± 1068.1	615.66 ± 1558.2	457.14 ± 274.2	44.01 ± 28.6	ns	ns	0.0027 **
Interferon stimulated genes	**PKR**	23.99 ± 34.3	16.43 ± 10.9	837.37 ± 2222.9	11.81 ± 7.4	26.83 ± 15.9	0.0001**	ns	ns
	**OAS**	15.61 ± 24.8	32.86 ± 33.1	785.82 ± 1158.7	21.19 ± 28.4	51.40 ± 65.6	0.0271*	ns	ns
	**MX1**	58.92 ± 14.1	104.76 ± 82.8	14702.68 ± 38494.2	283.59 ± 214.5	477.83 ± 168.3	0.0007**	ns	ns
	**MX2**	11.75 ± 10.1	10.45 ± 11.9	2258.53 ± 5784.8	22.17 ± 20.8	52.90 ± 23.3	<0.0001**	ns	ns
	**ISG12**	16.77 ± 19.1	73.45 ± 60.9	5583.01 ± 10106.2	869.02 ± 530.3	571.86 ± 265.8	ns	ns	ns
	**ISG15**	76.74 ± 78.6	204.13 ± 338.9	17267.75 ± 26798.8	182.57 ± 107.4	2508.50 ± 3556.5	0.0055**	ns	ns

Ns: not significant, SD: Standard deviation, ***P*<0.01 **P*<0.05, Genes showing significant differences in one of the conditions at least are represented in bold.
